# High salt-induced osmotic stress differentially modulates hepatocellular and renal carcinoma cell proliferation

**DOI:** 10.3389/fonc.2025.1693591

**Published:** 2026-01-09

**Authors:** Xi Chen, Asadur Rahman, Kento Kitada, Yaoyu Wang, Yoshihiro Nakajima, Changjin Liu, Rikiya Taoka, Akira Nishiyama

**Affiliations:** 1 Department of Pharmacology, Faculty of Medicine, Kagawa University, Miki-cho, Kagawa, Japan; 2 Health and Medical Research Institute, National Institute of Advanced Industrial Science and Technology (AIST), Takamatsu, Kagawa, Japan; 3 Department of Urology, Faculty of Medicine, Kagawa University, Miki-cho, Kagawa, Japan

**Keywords:** cell proliferation, hepatocellular carcinoma, high sodium, osmotic stress, renal carcinoma

## Abstract

**Introduction:**

High salt has been shown to affect cancer cell proliferation, however, its relationship with tumor growth remains controversial and mechanistically unclear. This study aims to elucidate how elevated sodium levels impact cell proliferation in hepatocellular carcinoma (HepG2) and renal carcinoma (Caki-1) cells, particularly under conditions of osmotic stress.

**Methods:**

Cells were exposed to high salt (50 mM NaCl above basal medium) and assessed their proliferation and the expression of NFAT5, a crucial osmoprotective transcription factor. The role of NFAT5 was further examined using targeted knockdown or overexpression in both cell lines. Xenograft models were also established using HepG2 or Caki-1 cells in immunocompromised mice, which were fed either a normal-salt (0.3% NaCl chow + tap water) or high-salt (4% NaCl chow + 1% NaCl water) diet.

**Results:**

High salt conditions led to a significant reduction in the proliferation of HepG2 cells both *in vitro* and *in vivo*, correlating with increased NFAT5 mRNA expression, while overexpression of NFAT5 effectively reversed this inhibition. Conversely, in Caki-1 cells, high salt did not significantly impact proliferation, tumor growth, or NFAT5 expression. However, NFAT5 knockdown in Caki-1 cells led to increased sensitivity and reduced proliferation in high-salt condition. Similar trends were observed with non-ionic osmoles (mannitol and sorbitol), which suppressed HepG2 cell proliferation without affecting Caki-1 cells.

**Conclusion:**

Taken together, these findings indicate the potential role of osmotic stress tolerance in the differential effects of hypertonic environments on HepG2 and Caki-1 cell proliferation, highlighting NFAT5 and associated osmoadaptive mechanisms as promising therapeutic targets within the tumor microenvironment.

## 1 Introduction

Sodium (Na^+^) accumulation in specific tissues and local microenvironments is increasingly recognized as a contributor to various pathological conditions. Notably, elevated dietary salt intake leads to an increase in sodium accumulation in the skin interstitium, which contributes to the development of salt-sensitive hypertension (1). Similarly, excessive salt consumption exacerbates tubulointerstitial inflammation and fibrosis in renal tissues, which can accelerate the progression of chronic kidney disease (2). Furthermore, hypernatremic conditions have been linked to the worsening of autoimmune diseases like multiple sclerosis, partly by promoting the differentiation of pro-inflammatory Th17 cells (3). The implications of local sodium enrichment also extend to oncology, where elevated sodium concentrations have been detected in tumor tissues via sodium magnetic resonance imaging (4). This phenomenon has been observed in various malignancies, including breast and prostate cancers, where the level of sodium accumulation correlates with the cancer aggressiveness (5, 6).

The functional significance of elevated sodium concentrations within the tumor microenvironment remains controversial. Extracellular sodium elevation triggers hyperosmotic stress, which can alter cancer cell behavior in a context-dependent manner (7). Notably, high-salt diets have been shown to suppress melanoma growth by increasing intratumoral sodium content (8). Similarly, in hepatocellular carcinoma (HCC) cells, elevated NaCl levels promote apoptosis and reduce viability *in vitro* (9). Mechanistic investigations further suggest that intracellular sodium accumulation in HCC induces energy depletion and cell death, thereby suppressing tumor growth *in vivo* (10). In contrast, epidemiological studies have linked high dietary salt intake to an increased risk of renal carcinoma (11, 12). Despite this elegant report, mechanistic insights regarding the response of renal cell carcinoma (RCC) cells to hyperosmotic stress are limited. Notably, different organs exhibit distinct physiological responses to osmotic stress. The kidney, which concentrates urine, is constantly exposed to high interstitial osmolarity, reaching up to 1,200 mOsm/kg H_2_O in humans and over 3,000 mOsm/kg H_2_O in rodents. This continuous exposure makes renal cells inherently more resistant to osmotic fluctuations (13). In contrast, the liver operates under relatively stable isotonic conditions and is not typically subject to significant osmotic variation. Consequently, hepatocytes are less adapted to osmotic stress, and hyperosmotic exposure can impair liver-specific metabolic and signaling functions (14).

Osmotic adaptation involves complex cellular processes involving various mechanisms, including the accumulation of compatible osmolytes and the activation of stress-responsive transcriptional pathways (7). A pivotal player in this adaptation is nuclear factor of activated T Cells (NFAT5), also known as tonicity-responsive enhancer-binding protein (TonEBP), which promotes the expression of osmolyte-related genes and supports cellular survival under hyperosmotic conditions. Upon hyperosmotic stress, intracellular ionic strength increases due to water efflux and subsequent cellular shrinkage. NFAT5 directly senses this ionic stress via its prion-like domain, which facilitates phase separation and the formation of nuclear condensates (15). These condensates subsequently recruit transcriptional cofactors, enabling the activation of osmoprotective genes, including the sodium/myo-inositol cotransporter and *aldose reductase* (16, 17). The upregulation of these genes facilitates the accumulation of compatible osmolytes like myo-inositol and sorbitol, aiding in the restoration of osmotic balance and enhancing cell survival under hyperosmotic conditions. Thus, NFAT5 acts as a crucial regulator of cellular responses to osmotic stress and serves as a potential molecular marker of osmotic imbalance in tissues (18). To better understand how tumor cells from different tissues respond to hyperosmotic stress, it is essential to examine the molecular mechanisms of osmotic adaptation.

Taken together, we hypothesize that the differential effects of high salt-induced osmotic stress on renal cell carcinoma (RCC) and HCC cells are primarily mediated by their intrinsic osmo-adaptive capacities. To test our hypothesis, this study aims to evaluate the impacts of high salt on the proliferation and tumor growth of both renal and HCC cells. Additionally, we examine the role of NFAT5 in contributing to the varying responses of these cell types, with the goal of elucidating the mechanisms governing tumor cell behavior under hyperosmotic conditions.

## 2 Materials and methods

### 2.1 Cell culture experiments

Human HCC (HepG2; RCB1886, RIKEN BRC, Tsukuba, Japan) and RCC (Caki-1; HTB-46, ATCC, MD, USA) cells were maintained in Dulbecco’s Modified Eagle Medium, high glucose (DMEM; 043-30085, FUJIFILM Wako Chemicals, Osaka, Japan), supplemented with 10% fetal bovine serum (FBS; 26140-079, Gibco, Thermo Fisher Scientific, MA, USA), 100 U/mL penicillin-streptomycin (15140122, Thermo Fisher Scientific), and 1X non-essential amino acids (11140050, Thermo Fisher Scientific), at 37 °C in a humidified incubator with 5% CO_2_. To induce hyperosmotic stress, NaCl (191-01665, FUJIFILM Wako Chemicals), D-mannitol (M4125-100G, Sigma-Aldrich, MO, USA), or D-sorbitol (198-03755, FUJIFILM Wako Chemicals) were added to the culture medium at specified concentrations. Cells were passaged at approximately 80% confluency using trypsin-EDTA (R00950, Thermo Fisher Scientific).

### 2.2 Cell proliferation assay

The water-soluble tetrazolium (WST)-1 assay was performed to assess cell proliferation, following the manufacturer’s protocol (MK400, Takara Bio, Shiga, Japan). Briefly, HepG2 or Caki-1 cells were seeded in 24-well plates at a density of 5 × 10^4^ cells/well and cultured under control or hyperosmotic conditions for 24, 48, or 72 hours. To induce hyperosmotic stress, the control medium (containing 110 mM NaCl) was supplemented with an additional 50 mM NaCl (final concentration 160 mM), 100 mM D-mannitol, or 100 mM D-sorbitol. At each time point, 50 μL of WST-1 reagent was added to each well, and the cells were incubated for 2 hours at 37 °C. Absorbance was measured at 440 nm using a microplate reader (SH-9000Lab, Corona Electric Co., Ltd., Ibaraki, Japan), and cell proliferation was expressed as a fold change relative to the control group.

### 2.3 NFAT5 knockdown and overexpression

HepG2 and Caki-1 cells were seeded at a density of 5 × 10^4^ cells/well in 24-well plates and cultured for 24 hours to reach approximately 50% confluency before transfection. For overexpression experiments, only HepG2 cells were used and seeded under the same conditions. NFAT5 knockdown was performed using ON-TARGETplus Human NFAT5 siRNA SMARTpool (L-009618-00-0050, Dharmacon, CO, USA) or ON-TARGETplus non-targeting pool (D-001810-10-50, Dharmacon) at a final concentration of 10 nM. Transfection was carried out using Lipofectamine™ RNAiMAX Transfection Reagent (13778075, Thermo Fisher Scientific), according to the manufacturer’s protocol. For NFAT5 overexpression, HepG2 cells were transfected with 500 ng/well of a codon-optimized human NFAT5 expression plasmid. The coding sequence was synthesized based on human codon usage, with a Kozak sequence added upstream of the start codon and removal of internal restriction enzyme sites. The optimized sequence was synthesized by GenScript (Piscataway, NJ, USA) and inserted into the *Hind*III and *Xba*I sites of the pcDNA3.1(+) mammalian expression vector (Invitrogen, CA, USA) under the control of a CMV promoter. The resulting construct (pcDNA3.1(+)-NFAT5) was verified by DNA sequencing. Transfection was performed using Lipofectamine^®^ LTX and Plus™ Reagents (15338030, Thermo Fisher Scientific), following the manufacturer’s instructions. After 24 hours, cells were washed and exposed to high NaCl stimulation (50 mM) or maintained under control conditions.

### 2.4 Real-Time quantitative PCR analysis

Total RNA was extracted using ISOGEN reagent (Nippon Gene, Tokyo, Japan) according to the manufacturer’s instructions, and cDNA was synthesized using the PrimeScript RT Reagent Kit (RR037A, Takara Bio). Quantitative PCR was performed on a 7300 Fast Real-Time PCR System (Applied Biosystems, Thermo Fisher Scientific) using power SYBR green PCR master mix (4367659, Thermo Fisher Scientific). NFAT5 expression and the expression of targeted osmolyte and ion homeostasis genes were measured using gene-specific primers listed in **Table 1**. Relative mRNA expression levels were calculated using the 2^−ΔΔCt method (19).

**Table 1:** Primer sequences used for Real-Time quantitative PCR.

Targeted gene	Forward	Reverse
*NFAT5*	AGAGTAGCGTTGAGGTTTGCT	GCATCCGGGTTATTCGGAGT
*SLC5A3*	GCCAGTACCATATTCACCCTCG	CATCTCCACGATGATTGGCACC
*AKR1B1*	CCAACTTCAACCATCTCCAGGTG	GTCACCACGATGCCTTTGGACT
*ATP1A1*	GGCAGTGTTTCAGGCTAACCAG	TCTCCTTCACGGAACCACAGCA
*SLC6A12*	GGAGAAACCTCGGGGCAT	AGGGCCAAAGCCAAGACAA
*18S*	CGTCACTTCTGGGGCCTTC	TTCTTGACACACCCACGG

### 2.5 Western blot analysis

Total cellular protein was extracted using RIPA-based lysis buffer supplemented with 1.0% Triton X-100 and a Protease Inhibitor Cocktail (04080-11, NACALAI TESQUE). Protein concentration was determined using the Pierce BCA Protein Assay Kit (23225, Thermo Fisher Scientific). Equal amounts of protein (50 μg) were separated by SDS-PAGE using Mini-PROTEAN TGX Gels (4561096, Bio-Rad) and transferred to PVDF membranes using iBlot 2 NC Regular Stacks (IB23001, Invitrogen by Thermo Fisher Scientific). The membranes were blocked with Intercept Blocking Buffer (927-70001, LI-COR) and incubated overnight at 4 °C with primary antibodies. Membranes were repeatedly washed with PBST (Phosphate-Buffered Saline containing 0.1% Tween 20). Then, membranes were incubated with the appropriate secondary antibodies, IRDye 680RD Goat anti-Mouse (926-68070, LI-COR) and IRDye 800CW Goat anti-Rabbit (926-32211, LI-COR). Protein bands were visualized using a LI-COR imaging system. Optical densities were quantified with ImageJ S5. Primary antibodies used include:NFAT5 (PA1-023, Invitrogen), p-ERK 1/2 (SC-136521, Santa Cruz Biotechnology), ERK 1/2 (SC-539888, Santa Cruz Biotechnology), HSP70 (10995-1-AP, Proteintech Group, Inc.), GAPDH (5174S, Cell Signaling Technology). Normalization Details: NFAT5, p-ERK 1/2, and ERK 1/2 expression in HepG2 cells was normalized to GAPDH. Due to observed variability of GAPDH expression under hyperosmotic stress in Caki-1 cells, normalization for Caki-1 was performed using HSP70.

### 2.6 Flow cytometry for intracellular sodium quantification

Intracellular Na^+^ was measured using ANG-AM, a sodium-sensitive fluorescent dye (ab142802, Abcam, Cambridge, UK). A stock solution (1.25 mM) of ANG-AM was prepared in DMSO containing 10% pluronic F-127 (59000, Biotium Inc., CA, USA), and diluted in culture medium to a final concentration of 0.25 μM ANG-AM and 0.02% pluronic F-127. Cells were incubated with the staining solution at 37 °C for 1 hour in a humidified 5% CO_2_ incubator. Fluorescence signals were analyzed using a CytoFLEX flow cytometer (Beckman Coulter, CA, USA) with excitation at 488 nm and detection at 585/42 nm (PE channel). A minimum of 20,000 cells per sample was acquired under identical instrument settings.

### 2.7 Transcriptomic and overall survival analysis using OncoDB

Transcriptomic data for *NFAT5* expressions, along with the associated clinical information, were extracted from the OncoDB database, which integrates RNA-seq, DNA methylation, and clinical information from TCGA and GTEx datasets (20). Both tumor and normal tissue samples were processed using a unified RNA-seq analysis pipeline recommended by the GDC. Briefly, raw reads were aligned to the human genome using STAR, followed by mapping to the RefSeq transcriptome to generate gene-level read counts. Counts were normalized using the transcripts per million (TPM) method to allow direct comparison between tissue types. Subsequently, we utilized the extracted clinical data to perform Overall Survival (OS) analysis for liver and kidney cancer patients. Patients were stratified into High expression and Low expression groups based on the NFAT5 TPM expression level. Kaplan-Meier curves were generated, and the Hazard Ratio (HR) and p-value were calculated to determine the prognostic value of *NFAT5* expression.

### 2.8 Animal experiments

All animal procedures were approved by the Animal Research Committee of Kagawa University (protocol numbers: 24635 and 24629). Male NOD/ShiJic-scid mice (Japan SLC Inc., Shizuoka, Japan), aged 6 weeks, were housed under controlled environmental conditions (temperature 24 ± 2 °C, humidity 55 ± 5%) with a 12-hour light/dark cycle. Animals had ad libitum access to food and water.

### 2.9 Xenograft tumor models

HepG2 or Caki-1 cells (2.5 × 10^6^ cells/mouse) were subcutaneously injected into the right flank of NOD/ShiJic-scid mice. For subcutaneous tumor implantation and tumor size measurement, mice were anesthetized using isoflurane inhalation (4–5% for induction, 2% for maintenance in oxygen) delivered via a precision vaporizer. Seven days after tumor implantation, the mice were randomly assigned to two groups: the control group received a normal salt diet (NSD) containing 0.3% NaCl and tap water, while a high-salt diet (HSD) group received 4% NaCl chow and 0.9% NaCl drinking water. Tumor volumes were measured weekly using calipers and calculated with the formula: Volume (mm³) = L × W²/2, where L is tumor length (mm) and W is tumor width (mm). At the end of the experiment (day 39), mice were euthanized by cervical dislocation under deep anesthesia induced with isoflurane. Tumors were excised and processed for further analysis.

### 2.10 Statistical analysis

Data are presented as mean ± standard error of the mean (SEM) from at least three independent experiments (N ≥ 3). For comparisons between two groups, unpaired t-tests were performed. For multiple-group comparisons, either one-way or two-way ANOVA was applied, depending on the experimental design, followed by Tukey’s *post hoc* test. A p-value < 0.05 was considered statistically significant. Statistical analyses were conducted using GraphPad Prism 8.0.1 software (GraphPad Software, CA, USA).

## 3 Results

### 3.1 High salt suppressed HepG2 cell proliferation and xenograft tumor growth

**Figure 1:**
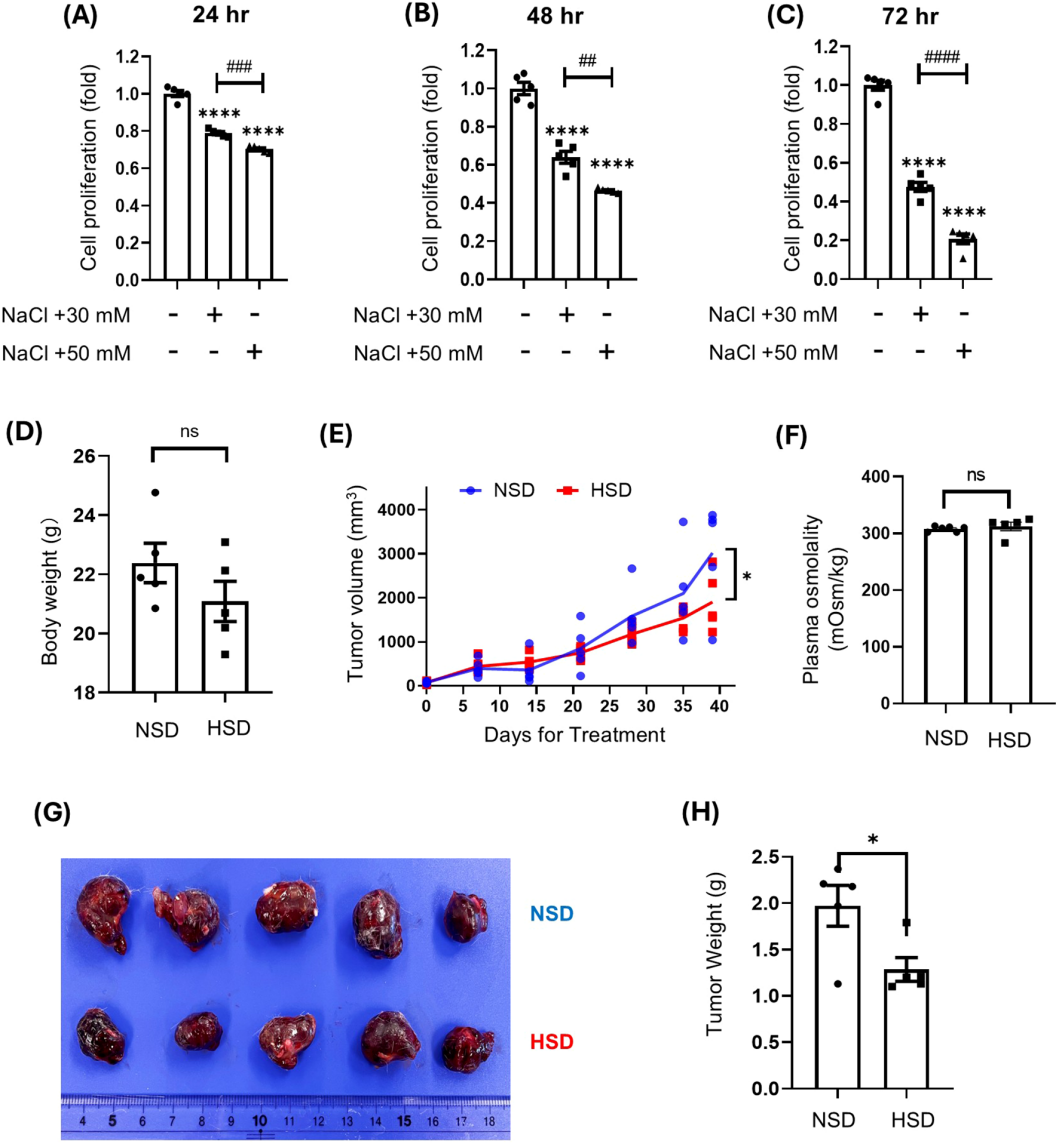
Effects of high salt on HepG2 cell proliferation *in vitro* and tumor growth *in vivo.***(A–C)** HepG2 cells were treated with 30 or 50 mM NaCl for 24 h **(A)**, 48 h **(B)**, or 72 h **(C)**. Proliferation was measured by WST-1 assay. The control group represents standard DMEM containing 110 mM NaCl, while the treatment groups reflect additional NaCl supplementation beyond this baseline. **(D–H)** HepG2 xenografts were established in NOD/ShiJic-scid mice and treated with either a normal-salt diet (NSD; 0.3% NaCl chow and tap water) or a high-salt diet (HSD; 4% NaCl chow and 0.9% NaCl water). body weight **(D)**. Tumor volumes were monitored over 39 days **(E)**. Plasma osmolality was measured at the study endpoint in mice from the NSD and HSD groups **(G)**. Representative tumors at endpoint are shown **(G)** and Tumor weight **(H)** were recorded. Data are presented as mean± SEM (n≥ 5 per group). *p < 0.05, ****p < 0.0001 vs. control group; ##*p* < 0.001 ###*p* < 0.001, *p* < 0.0001 vs. NaCl +30 mM treatment. Data are presented as mean± SEM (n≥ 5 per group). Statistical analysis was performed using one-way ANOVA.

High concentrations of NaCl suppressed HepG2 cell proliferation in a dose- and time-dependent manner (**Figures 1A–C**). Both 30 mM and 50 mM NaCl significantly reduced proliferation at all time points, with the strongest inhibition observed at 72 hours under 50 mM NaCl treatment, where cell viability dropped to approximately 20% of the control level (**Figure 1C**). Mouse body weight remained unchanged across both dietary conditions throughout the study period (**Figure 1D**). While HepG2 xenograft tumors grew progressively over time in both NSD or HSD groups, the tumor growth rate was significantly lower in the HSD group from day 20 onwards than that in the NSD group (**Figure 1E**). Furthermore, measurement of plasma osmolality at the study endpoint showed no significant difference between the NSD and HSD groups (**Figure 1F**). By day 37, xenograft tumor volume was significantly smaller in the HSD group compared to the control group (**Figures 1G, H**).

### 3.2 High salt did not alter Caki-1 cell proliferation and xenograft tumor growth

**Figure 2:**
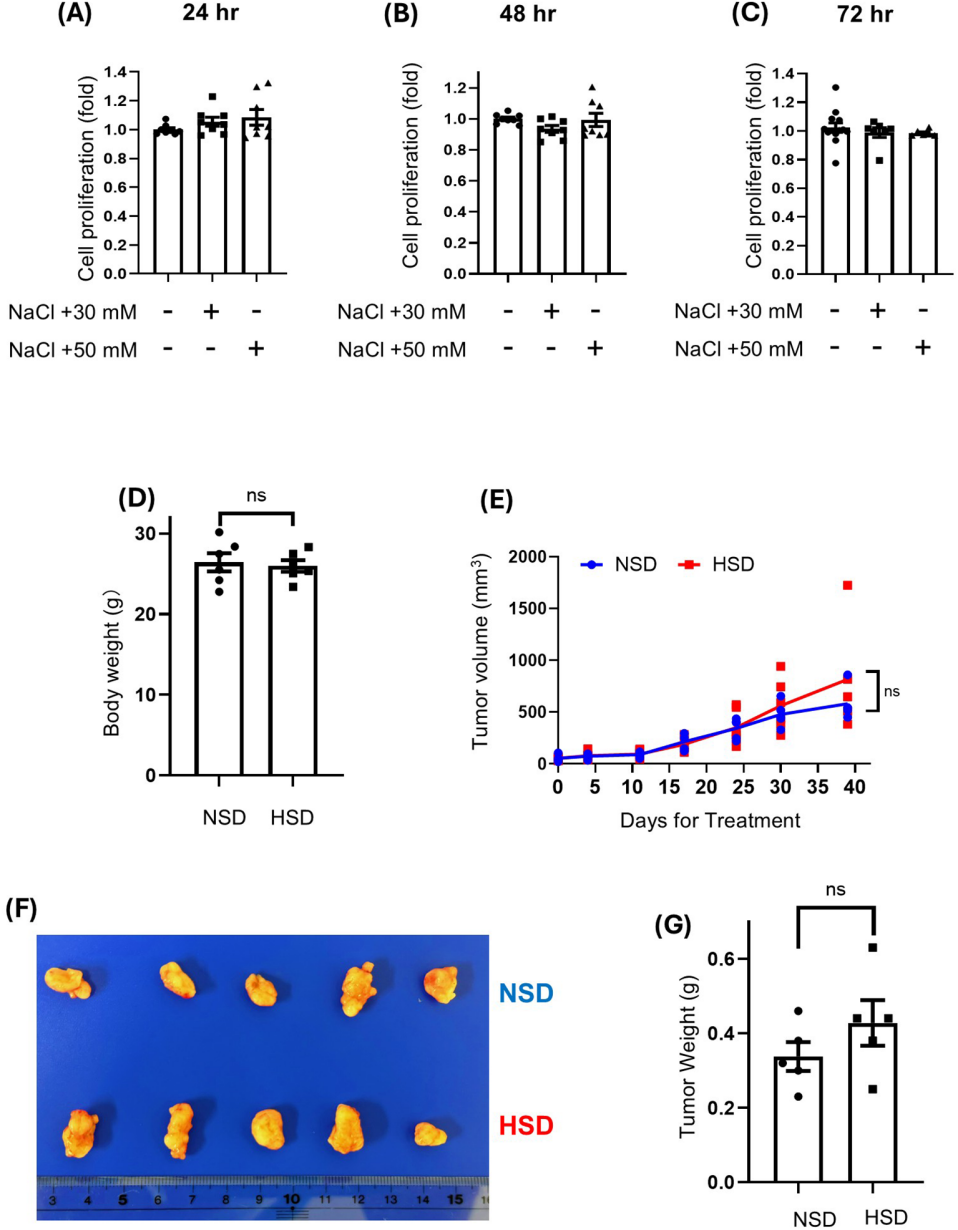
Effects of high-salt conditions on Caki-1 cell proliferation *in vitro* and tumor growth *in vivo.***(A-C)** Caki-1 cells were treated with 30 mM or 50 mM NaCl for 24 h **(A)**, 48 h **(B)**, or 72 h **(C)**. Cell proliferation was assessed using the WST-1 assay. The control group represents standard DMEM containing 110 mM NaCl, while the treatment groups reflect additional NaCl supplementation beyond this baseline. **(D–G)** Caki-1 xenografts were established in NOD/ShiJic-scid mice and treated with either a normal-salt diet (NSD; 0.3% NaCl chow and tap water) or a high-salt diet (HSD; 4% NaCl chow and 0.9% NaCl water). body weight **(D)**. Tumor volumes were monitored weekly for 39 days **(E)**. Representative tumors are shown **(F)**. Tumor weight was recorded at the endpoint **(G)**. ns = not significant. Data are presented as mean± SEM (n≥ 5 per group). Statistical analysis was performed using one-way ANOVA.

High concentration of NaCl exposure did not alter Caki-1 cell proliferation, with similar growth curves observed across all tested concentrations and time points (**Figures 2A–C**). Mouse body weight remained unchanged across both dietary conditions throughout the study (**Figure 2D**). Furthermore, HSD did not significantly impact Caki-1 xenograft tumor growth. No significant differences were observed in tumor size or weight between the NSD and HSD groups (**Figures 2E–G**). While tumors in the HSD group were marginally larger than those in the NSD, this difference was not statistically significant. To further investigate the differential responses between HepG2 and Caki-1 cells under hyperosmotic conditions, we measured intracellular sodium levels using the sodium-sensitive fluorescent dye ANG-AM. Flow cytometry histograms (**Supplementary Figure 1A**) and their corresponding quantification (**Supplementary Figure 1B**) revealed that Caki-1 cells exhibited significantly greater ANG-positive cell populations than HepG2 cells following +50 mM NaCl exposure. As Caki-1 cells showed increased intracellular Na^+^ accumulation under hyperosmotic stress, we analyzed the expression of genes encoding related ion channels and pumps. qPCR analysis showed that 50 mM NaCl exposure significantly reduced *ATP1A1* mRNA expression (**Supplementary Figure 2A**). Similarly, *TRPM4* mRNA expression was significantly decreased by 50 mM NaCl treatment (**Supplementary Figure 2B**).

### 3.3 *In vitro* and *in vivo* expression of NFAT5 mRNA

**Figure 3:**
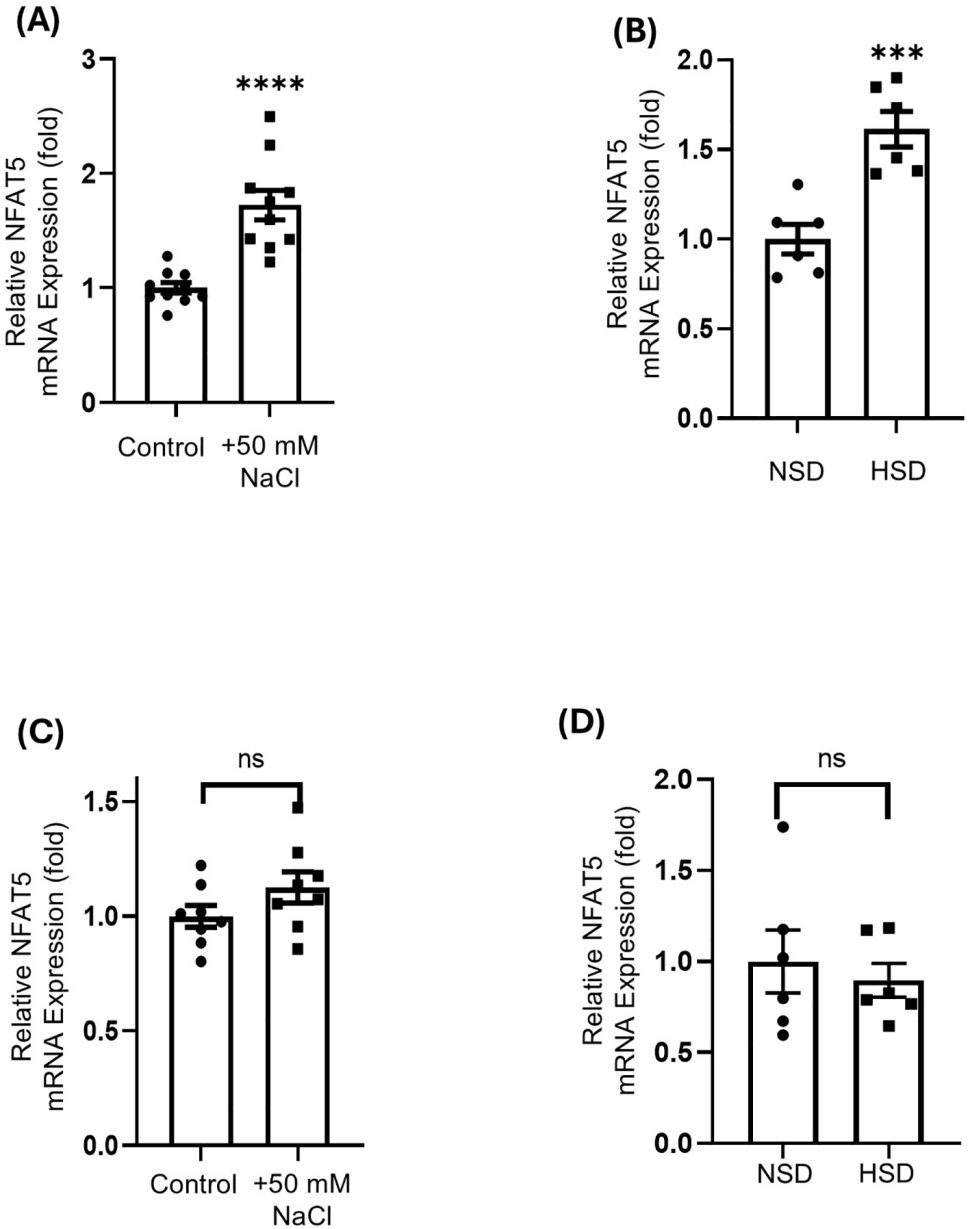
Relative NFAT5 mRNA expression in HepG2 and Caki-1 cells under high-NaCl conditions *in vitro* and *in vivo.***(A)** NFAT5 mRNA expression in HepG2 cells cultured with or without 50 mM NaCl, **(B)** and in HepG2 xenograft tumors from mice fed a normal-salt diet (NSD) or high-salt diet (HSD). **(C)** NFAT5 mRNA expression in Caki-1 cells under the same *in vitro***(D)** and *in vivo* conditions as in **(A, B)**. *In vitro* control medium contains 110 mM NaCl, while the treatment groups represent additional NaCl supplementation. ***P < 0.001 **** *P* < 0.0001 vs. control or NSD group. Data are presented as mean± SEM (n≥ 5 per group). Statistical analysis was performed using one-way ANOVA.

To investigate the cellular response to hyperosmotic stress, we assessed *NFAT5* mRNA expression in both HepG2 and Caki-1 cell lines. In HepG2 cells, high salt treatment significantly induced *NFAT5* mRNA expression, demonstrating a 1.7-fold increase *in vitro* (**Figure 3A**) and a 1.6-fold increase *in vivo* (**Figure 3B**), compared to the control groups. Conversely, Caki-1 cells showed no significant differences in *NFAT5* mRNA expression in response to high salt in either *in vitro* (**Figure 3C**) or *in vivo* (**Figure 3D**) models. These findings suggest that HepG2 cells are osmotically sensitive to high salt, while Caki-1 cells exhibit resistance. Supporting these results, analysis of publicly available transcriptomic data via the OncoDB platform revealed that *NFAT5* expression is markedly higher in both normal and cancerous kidney tissues than in liver tissues (**Supplementary Figure 3**), indicating potential tissue-specific differences in *NFAT5* expression. Furthermore, Overall Survival (OS) analysis using OncoDB data revealed differential prognostic value of *NFAT5* in liver and kidney cancer (**Supplementary Figure 4**). In Liver cancer, high *NFAT5* expression showed no significant correlation with OS (*p* = 0.43; HR = 1.15 [0.81-1.63]). In Kidney cancer, high *NFAT5* expression was significantly associated with better OS (*p* = 0.01; HR = 0.67 [0.50-0.91]), suggesting a protective role of *NFAT5* in kidney tumor patients.

### 3.4 Impact of NFAT5 modulation on HepG2 and Caki-1 cells

**Figure 4:**
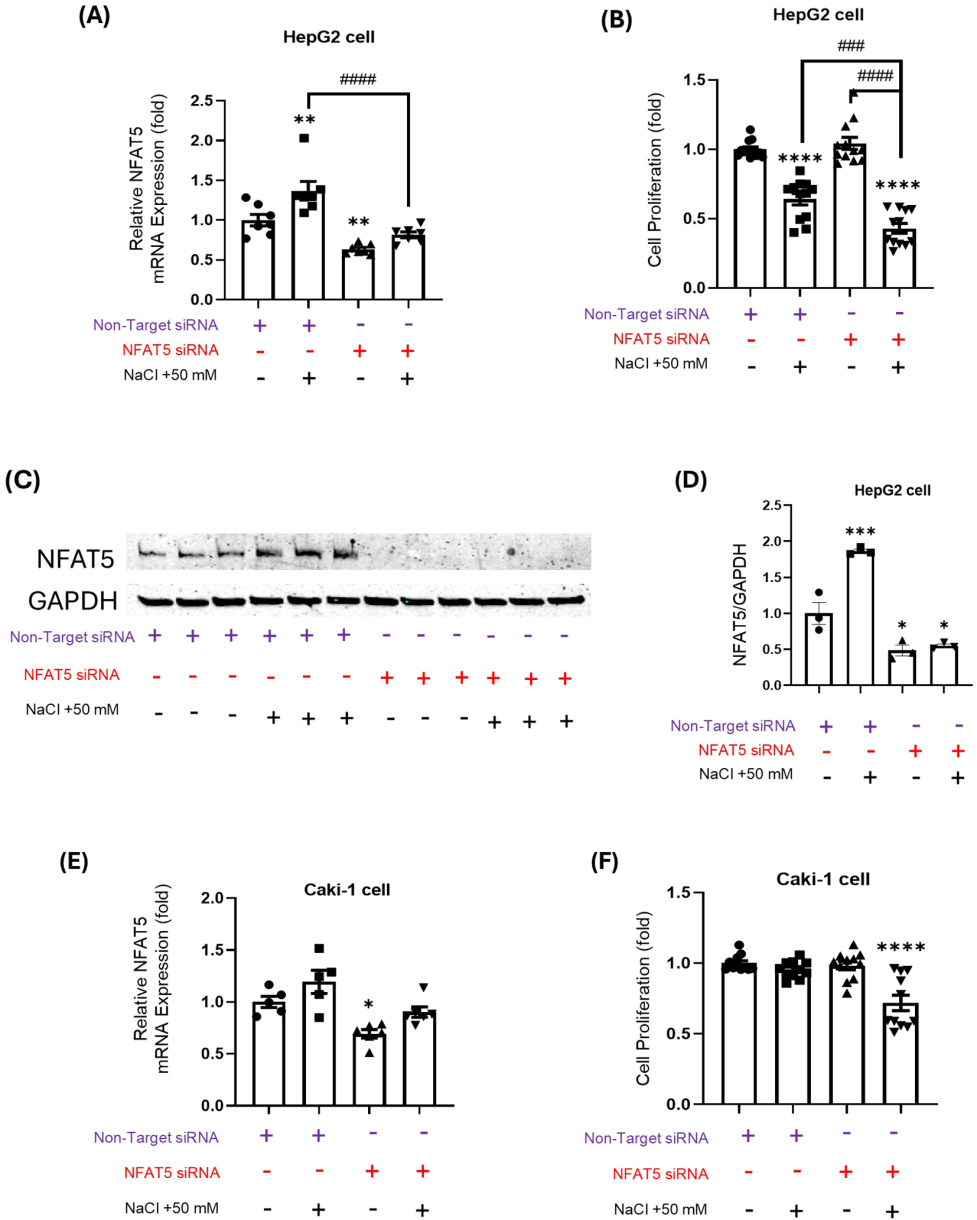
Effects of NFAT5 knockdown on HepG2 and Caki-1 cell responses under high-NaCl conditions. **(A, B)** Relative NFAT5 mRNA expression **(A)** and cell proliferation **(B)** in HepG2 cells under the following conditions: control, 50 mM NaCl, NFAT5 siRNA, or NFAT5 siRNA + 50 mM NaCl. **(C)** Western Blot analysis of NFAT5 protein expression in HepG2 cells following transfection with NFAT5 siRNA and/or culture with 50 mM NaCl. **(D)** Relative NFAT5 mRNA expression in HepG2 cells based on the Western Blot NFAT5 band intensity quantification from **(C)** (normalized to GAPDH). **(E, F)** Relative NFAT5 mRNA expression **(E)** and cell proliferation **(F)** in Caki-1 cells under the same four conditions. *P < 0.05, **P < 0.01, ****P < 0.0001 vs. control or non-target siRNA group; ###*P* < 0.001, ####*P* < 0.0001 vs. NFAT5 siRNA + 50 mM NaCl group. ns = not significant. Data are presented as mean± SEM (n≥ 3 per group). Statistical analysis was performed using one-way ANOVA.

In HepG2 cells, the application of *NFAT5* siRNA resulted in a significant reduction of *NFAT5* mRNA levels in both control and high salt-treated cells (**Figure 4A**). This knockdown efficacy was further validated by Western blot analysis, which showed a significant decrease in NFAT5 protein levels in the NFAT5 siRNA group compared to the non-target siRNA control group (**Figures 4C, D**). This *NFAT5* knockdown, which enhances hyperosmotic stress, led to a further decrease in cell proliferation in high salt-treated HepG2 cells compared to the control group (**Figure 4B**). These findings suggest that NFAT5 has a protective role in HepG2 cells under hyperosmotic stress. Similarly, in Caki-1 cells, NFAT5 siRNA effectively suppressed *NFAT5* mRNA expression (**Figure 4E**). Notably, only *NFAT5*-knockdown in Caki-1 cells showed a marked decrease in proliferation under high salt conditions, whereas control siRNA-transfected cells remained unaffected (**Figure 4F**). Overall, these results indicate that NFAT5 contributes to cellular survival in both cell types; however, the specific requirement for NFAT5 upregulation might differ depending on the inherent tolerance of each cell types to osmotic changes.

**Figure 5:**
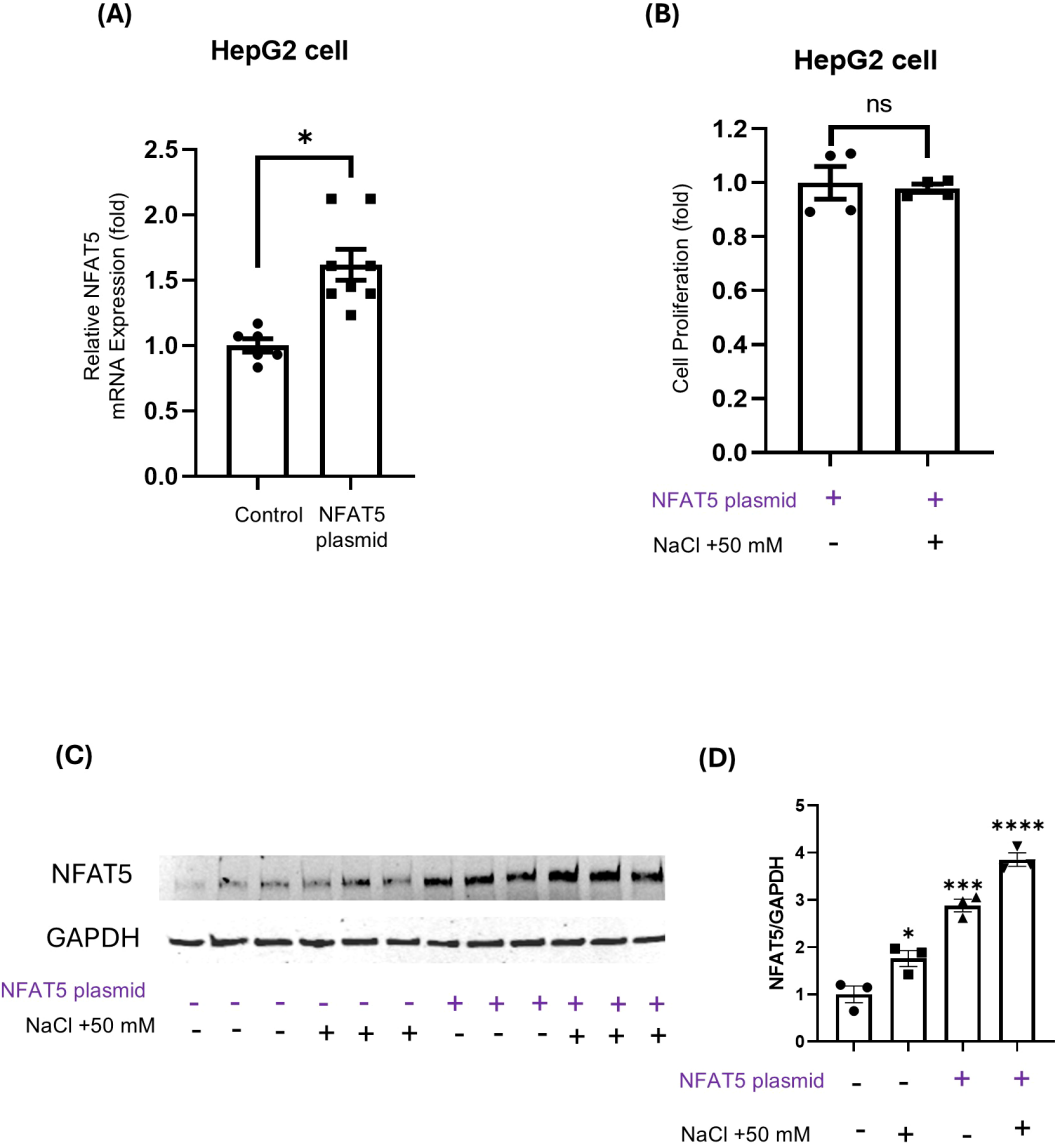
Effects of NFAT5 overexpression on HepG2 cells. **(A, B)** Relative NFAT5 mRNA expression **(A)** and cell proliferation **(B)** in HepG2 cells transfected with an NFAT5 overexpression plasmid, with or without 50 mM NaCl. (C) Western Blot analysis of NFAT5 protein expression in HepG2 cells following transfection with NFAT5 plasmid and/or culture with 50 mM NaCl. (D) Quantification of NFAT5 protein expression from **(C)** (normalized to GAPDH). *P < 0.05, ***P < 0.001, ****P < 0.0001; ns = not significant. Comparisons were made against the control group **(A)**, the NFAT5 plasmid (+) / NaCl (−) group **(B)**, or the NFAT5 plasmid (−) / NaCl (−) group **(D)**. Data are presented as mean± SEM (n≥ 3 per group). Statistical analysis was performed using one-way ANOVA (D) or unpaired t-test **(A, B)**.

To further understand the role of NFAT5 in HepG2 cells, we next examined the effects of *NFAT5* overexpression. NFAT5 plasmid transfection significantly increased *NFAT5* mRNA levels (**Figure 5A**). This successful overexpression was confirmed at the protein level by Western blot analysis, which demonstrated a marked increase in NFAT5 protein expression in the *NFAT5* plasmid group compared to the control group (**Figures 5C, D**). Crucially, high salt-induced suppression of cell proliferation was no longer observed in NFAT5-overexpressing HepG2 cells (**Figure 5B**). These findings support the idea that enhancing NFAT5 expression is sufficient to confer osmotic protection in sensitive cell types like HepG2.

### 3.5 NFAT5 regulates osmoprotective gene expression

NFAT5 modulation revealed opposing regulatory mechanisms between the two cell lines (**Supplementary Figures 5**-**7**). In HepG2 cells: *NFAT5* siRNA significantly suppressed the high salt-induced upregulation of *SLC5A3*, *SLC6A12*, and *AKR1B1* (**Supplementary Figures 5A–C**). *NFAT5* overexpression further potentiated this salt-induced upregulation (**Supplementary Figures 6A–C**). This confirms NFAT5 functions as a stress-inducible transcriptional activator in HepG2. In Caki-1 cells: NFAT5 siRNA knockdown caused a significant increase in the basal mRNA expression of *SLC5A3* and *AKR1B1* (**Supplementary Figures 7A, C**), suggesting *NFAT5* acts as a transcriptional repressor in the basal state. Furthermore, high salt alone significantly suppressed the expression of these genes. When *NFAT5* siRNA was combined with high salt, the gene expression levels were returned to the highly suppressed level seen in the high salt-only group. This demonstrated that the high salt-induced suppression effect overrides the NFAT5 basal repression signal.

### 3.6 Differential sensitivity of HepG2 and Caki-1 cells to osmotic stress mediated by various osmole

**Figure 6:**
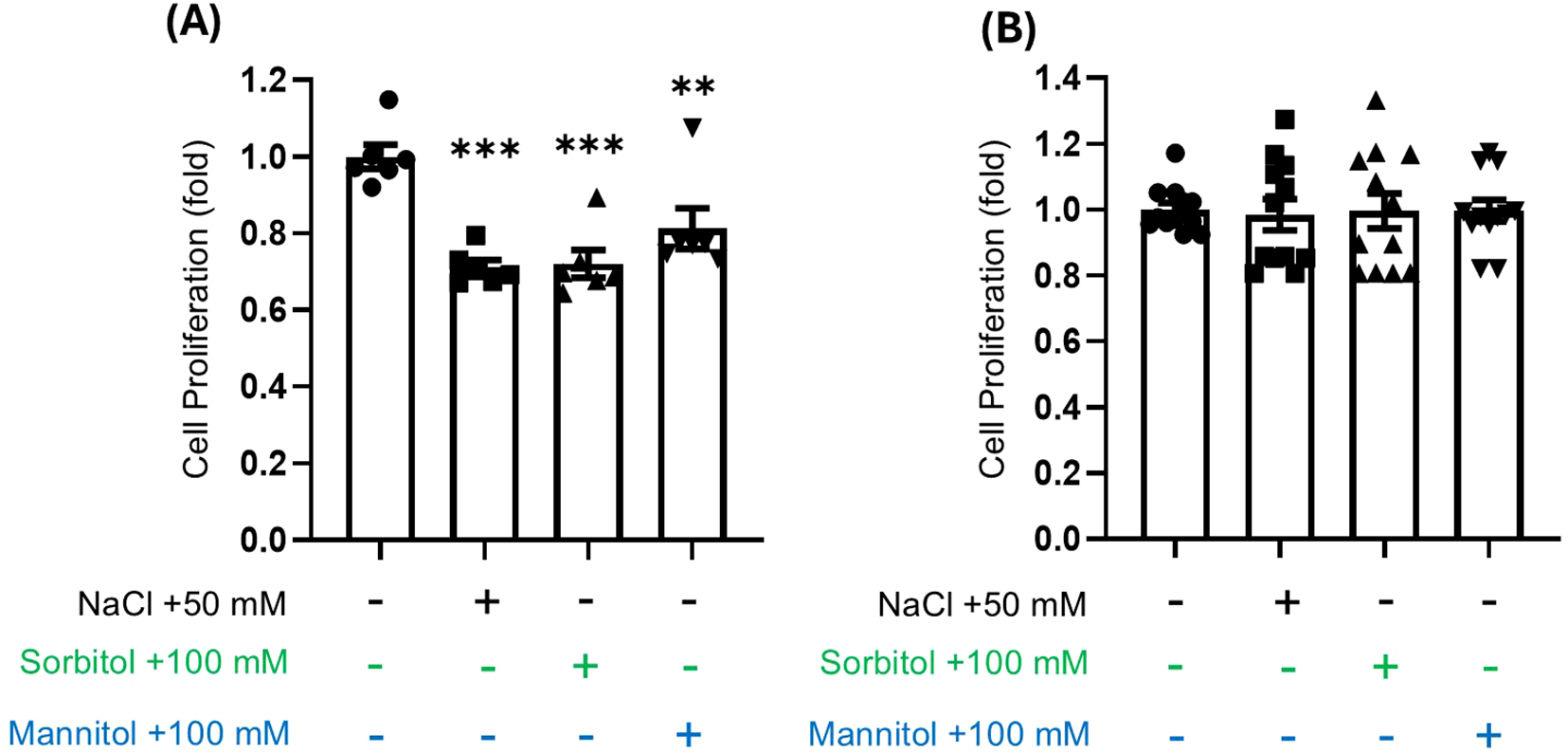
Effects of different osmolytes on HepG2 and Caki-1 cell proliferation at equivalent osmolarity. **(A)** HepG2 and **(B)** Caki-1 cells were cultured under iso-osmotic conditions (+50 mM NaCl, +100 mM sorbitol, or +100 mM mannitol) for 48 h. Cell proliferation was assessed by WST-1 assay. **P < 0.01, ***P < 0.001 vs. control group, ns = not significant. Data are presented as mean± SEM (n≥ 6 per group). Statistical analysis was performed using one-way ANOVA.

To determine whether the observed effects of NaCl on cell proliferation were unique to sodium or indicative of a broader response to osmotic stress, we investigated the impact of mannitol, and sorbitol on the proliferation of both HepG2 and Caki-1 cells. Our results demonstrated that HepG2 cell proliferation was significantly inhibited by all tested osmoles (**Figure 6A**), whereas Caki-1 cells exhibited no change in proliferation under similar conditions (**Figure 6B**). To further evaluate osmotic stress–responsive pathways, we measured HSP70 and ERK1/2 phosphorylation. In HepG2 cells, HSP70 levels were unchanged, whereas NaCl consistently increased ERK1/2 phosphorylation regardless of NFAT5 knockdown or overexpression (**Supplementary Figures 8A–C**, **9A–C**). In Caki-1 cells, neither HSP70 nor ERK1/2 phosphorylation showed appreciable changes under any condition (**Supplementary Figures 8D, E**). These results indicate that HepG2, but not Caki-1, cells exhibit ERK1/2 activation in response to hypertonic stress.

## 4 Discussion

High sodium levels have been reported to influence cancer progression (8, 9); however, their effects can vary considerably depending on the tumor context. In this study, we demonstrated that HepG2 and Caki-1 cells exhibited differential sensitivity to high salt exposure regarding cell proliferation, both *in vitro* and *in vivo*. Specifically, HepG2 cells showed marked growth inhibition under hyperosmotic conditions, whereas Caki-1 cells remained largely resistant. Although high-salt diets can raise potential systemic confounders, our measurement of plasma osmolality showed no significant difference between the dietary groups, suggesting the tumor effect is mediated by localized osmotic stress rather than systemic hyperosmolarity. These differences correlated with the expression of NFAT5, which was upregulated in HepG2 cells but unchanged in Caki-1 cells. Furthermore, NFAT5 knockdown enhanced salt sensitivity in both cell lines, while its overexpression restored proliferation in HepG2 cells. These findings suggest that variations in NFAT5-mediated osmoadaptive capacity might explain the divergent effects of high salt on cancer cell proliferation.

Different organs exhibit varying sensitivity or tolerance to hyperosmotic stress, a phenomenon primarily determined by their physiological functions and native osmotic environments. This distinction may explain the observed higher salt sensitivity in HepG2 cells compared to Caki-1 cells, consistent with previous reports on the osmotic vulnerability of hepatic tissues and the relative osmoprotective nature of renal cells (10, 11).

NFAT5 serves as a principal transcriptional regulator that enables cells to adapt to hyperosmotic environments. Upon osmotic stress, NFAT5 promotes the transcription of osmoprotective genes, such as *SMIT1* and *aldose reductase*. This activation leads to the intracellular accumulation of compatible osmolytes, which helps to prevent cell shrinkage or death (21, 22). Notably, human transcriptomic data revealed that NFAT5 expression is markedly elevated in the kidney compared to the liver, both in normal and cancerous states (23, 24). This pattern aligns with the kidney’s adaptation to inherently hyperosmotic conditions. In our study, HepG2 cells exhibited marked upregulation of NFAT5 mRNA upon exposure to elevated NaCl levels, both *in vitro* and *in vivo*. In contrast, Caki-1 cells, which are of renal origin, did not exhibit significant changes in NFAT5 expression. This lack of change likely reflects the sufficient baseline levels of NFAT5 already present in these cells due to their inherent adaptation to osmotic stress. These findings imply that liver-derived cancer cells require NFAT5 upregulation to cope with hyperosmotic stress effectively, whereas kidney-derived cancer cells inherently possess the capacity to withstand such conditions without necessitating additional activation of NFAT5. Our findings on the differential regulation of *NFAT5* are further supported by *in vivo* studies. Specifically, Sprague–Dawley rats fed an 8% NaCl diet for eight weeks showed a dramatic (~55-fold) increase in NFAT5 mRNA in the liver, while only a modest elevation was observed in the kidney medulla. These results reinforce the notion that hepatic tissues require greater NFAT5 induction to adapt to osmotic stress, unlike renal tissues, which are already osmotically conditioned (25).

Our data revealed that NFAT5 knockdown significantly reduced proliferation in both HepG2 and Caki-1 cells under high salt conditions. Conversely, overexpression of NFAT5 successfully rescued growth in HepG2 cells under similar stress. These functional findings align with NFAT5’s established role in renal osmoadaptation (26), as evidenced by studies showing that mice lacking NFAT5 develop severe renal atrophy and die shortly after birth (27). Collectively, our results, supported by prior evidence, underscore the essential role of NFAT*5* in promoting cell viability under hyperosmotic conditions, a critical function observed in both normal renal tissue and cancer cells.

The observed tissue-specific regulation of NFAT5 prompted us to further investigate its functional relevance in response to osmotic stress. We assessed the expression of known osmolyte transporters, *SLC5A3* (*SMIT1*), *SLC6A12* (*BGT1*), and *AKR1B1* (*aldose reductase*), to dissect the downstream effects of NFAT5. In HepG2 cells, high salt significantly induced the mRNA of all three osmolyte genes. Crucially, NFAT5 overexpression further enhanced this induction, while NFAT5 siRNA knockdown significantly reduced the hyperosmotic induction. These findings confirm NFAT5’s role as an essential positive regulator for osmoadaptive gene expression in HepG2 cells, consistent with its canonical function (7). Conversely, Caki-1 cells demonstrated a critical deviation from this dogma, revealing a novel repressive function for NFAT5. High salt primarily suppressed the expression of these osmolyte genes. Crucially, NFAT5 knockdown alone resulted in a dramatic increase their basal expression, indicating that in this specific renal cancer context, NFAT5 functions as a transcriptional repressor of these metabolic genes. This functional transition aligns with studies showing NFAT5 regulation is highly context-dependent (28) and can involve both activating and repressive functions across different cell lineages (29). This observed switch to a repressive function provides a mechanistic explanation for our key clinical finding: high *NFAT5* expression correlates with significantly improved survival in kidney cancer patients. NFAT5 likely acts as a protective marker by serving as a transcriptional repressor to control the aberrant expression of metabolic pathways (28, 29). Specifically, the loss of this repression (low NFAT5) drives the subsequent aggressive phenotype seen in tumors by causing the uncontrolled increase of *AKR1B1* (30; Luojie 31).

Beyond NFAT5-dependent mechanisms, ion handling may also contribute to osmoadaptive capacity. Interestingly, Caki-1 cells accumulated significantly more intracellular Na^+^ than HepG2 cells after 24 hours of exposure to 50 mM NaCl, suggesting a mechanism that may actively facilitate cation influx to combat the external hyperosmotic pressure. To investigate this, we analyzed *ATP1A1* (a subunit of the Na^+^/K^+^-ATPase) and *TRPM4* expression. We observed that *ATP1A1* mRNA and *TRPM4* expressions were significantly downregulated in Caki-1 cells following high-salt exposure. This Na^+^ accumulation in Caki-1 cells, along with the downregulated expression of *ATP1A1* and TRPM4, suggests a regulated shift in cellular Na^+^ flux supporting a volume regulatory strategy, which is consistent with Na^+^ ‘s known role in preserving cell volume and regulating ASK3 via *TRPM4* channel activity (32). In contrast, HepG2 but not Caki-1 cell proliferation was inhibited by the tested osmoles, sorbitol and mannitol. This indicated that the observed growth suppression might be primarily driven by osmotic pressure rather than ion-specific toxicity. Such adaptive mechanisms help prevent cytoplasmic crowding and maintain critical biochemical signaling pathways necessary for cell survival and proliferation (7).

To determine whether NFAT5 modulates general stress pathways beyond osmoadaptation, we examined MAPK signaling and HSP70 expression. In HepG2 cells, hypertonicity decreased total ERK1/2 while enhancing its phosphorylation—an established feature of stress-amplified MAPK activation in hyperosmotic conditions (33, 34). These changes occurred independently of NFAT5 levels, indicating a general osmotic stress response rather than NFAT5-mediated signaling. In contrast, Caki-1 cells showed minimal alterations in ERK1/2 or HSP70, consistent with the well-recognized osmotic tolerance of renal-derived epithelial cells that are naturally adapted to high-osmolarity environments. These findings indicate that NFAT5 modulation does not broadly alter MAPK or HSP70 signaling, supporting the conclusion that its primary function in these cells is osmotic adaptation rather than activation of general stress pathways.

This study has several limitations that warrant consideration. First, HepG2 cells are hepatoblastoma-derived rather than adult HCC and therefore may not fully represent the biological characteristics of adult HCC. In addition, while our results suggest that differences in osmotic stress tolerance and regulation of NFAT5 contribute to the variable salt sensitivity observed in HepG2 and Caki-1 cells, the precise molecular mechanisms underlying these differences remain unclear. Further investigations are necessary to identify the upstream and downstream regulators associated with this response. Second, all experiments were conducted in systems comprising only cancer cells, without considering the role of immune cells. Given that tissue sodium accumulation can influence immune function and the efficacy of anti-tumor responses (35), our *in vitro* and xenograft models may not fully reflect the complexity of *in vivo* environments. Specifically, alterations in blood pressure, could still contribute to the *in vivo* outcomes. Finally, the supplementation with artificial osmolytes may not accurately replicate the tumor microenvironment. Future work should incorporate more physiologically relevant systems, such as models that include a broader panel of adult HCC cell lines, immune-competent cells or those derived from patient samples. Despite these limitations, our findings underscore the critical role of osmotic stress tolerance in cancer cell behavior and support the need for further exploration of NFAT5 and related osmo-adaptive pathways as potential therapeutic targets.

In summary, our study emphasizes that osmotic stress tolerance is a critical determinant of cancer cell proliferation in high-salt environments. The contrasting responses observed in HepG2 and Caki-1 cells likely reflect tissue-specific variations in osmo-adaptive capacity, particularly regarding the regulation of NFAT5. These findings establish a foundational basis for future investigations focused on targeting osmotic vulnerability in tumors by modulating stress-adaptive pathways.

## Data Availability

The original contributions presented in the study are included in the article/**Supplementary Material**. Further inquiries can be directed to the corresponding author.
