# Supplementary material for: High salt-induced osmotic stress differentially modulates hepatocellular and renal carcinoma cell proliferation
Source: Front Oncol. 2026 Jan 9;15:1693591. doi: 10.3389/fonc.2025.1693591 (PMC12827174; doi:10.3389/fonc.2025.1693591)
Supplement: Supplementary file 1 [file DataSheet1.docx]

**Supplementary Figure 1: Intracellular Na⁺ Accumulation in HepG2 and Caki-1 Cells After High-NaCl Exposure.**

(A) Representative flow cytometry histograms of ANG-PE fluorescence intensity in HepG2 (top) and Caki-1 (bottom) cells under control and 50 mM NaCl conditions for 24 hours. (B) Quantification of ANG-PE⁺ cell percentages in HepG2 and Caki-1 cells. ** *P* < 0.01 vs. control group. Data are presented as mean± SEM (n= 3 per group). Statistical analysis was performed using two-way ANOVA.

**Supplementary Figure 2. Relative ATP1K1and TRPM4 mRNA expression in Caki-1 cells under high-NaCl conditions.**

(A) Relative ATP1K1 mRNA expression in Caki-1 cells cultured with or without 50 mM NaCl. (B) Relative TRPM4 mRNA expression in Caki-1 cells under the same in vitro conditions as in (A). **P* < 0.05, ***P* < 0.01 vs. control group; ns = not significant. For (A) and (B), data are presented as mean ±SEM (n=6). In vitro control medium contains 110 mM NaCl, while the high-NaCl treatment groups represent additional NaCl supplementation. Statistical analysis was performed using t-test.

**Supplementary Figure 3: NFAT5 mRNA expression in kidney and liver tissues based on TCGA and GTEx datasets.**

Expression levels were extracted from the OncoDB database. Data are presented as TPM-normalized values across normal liver hepatocellular carcinoma (HCC), normal kidney, and renal cell carcinoma (RCC), normal kidney, samples. NFAT5 mRNA expression was significantly higher in kidney tissues (both tumor and normal) than in liver tissues. *****P* < 0.0001 vs. KIRC group. Data are presented as mean ±SEM (n≥44). Statistical analysis was performed using two-way ANOVA.

**Supplementary Figure 4. Overall survival analysis based on NFAT5 mRNA expression in liver cancer and kidney cancer tissues based on TCGA and GTEx data.**

(A) Kaplan-Meier overall survival curves for patients with Liver Cancer stratified by the expression level of NFAT5 (High expression, n=186; Low expression, n=185). The *P* value is 0.43, with a Hazard Ratio (HR) of 1.15 (0.81-1.63). (B) Kaplan-Meier overall survival curves for patients with Kidney Cancer stratified by the expression level of NFAT5 (High expression, n=266; Low expression, n=266). The *P* value is 0.01, with a Hazard Ratio (HR) of 0.67 (0.50-0.91).

**Supplementary Figure 5. Relative NFAT5 target gene mRNA expression in HepG2 cells under NFAT5 modulation and high-NaCl conditions.**

(A) Relative SLC5A3 mRNA expression in HepG2 cells following transfection with NFAT5 siRNA and/or culture with 50 mM NaCl. (B) Relative SLC6A12 mRNA expression in HepG2 cells under the same in vitro conditions as in (A). (C) Relative AKR1B1 mRNA expression in HepG2 cells under the same in vitro conditions as in (A). **P*< 0.05 vs. nogroup; siRNA control group; #*P* < 0.05 vs. NFAT5 siRNA + 50 mM NaCl group. ns = not significant. For (A), (B), and (C), data are presented as mean SEM (n=3 biological replicates). In vitro control medium contains 110 mM NaCl, while the high-NaCl treatment groups represent additional NaCl supplementation. Statistical analysis was performed using one-way ANOVA.

**Supplementary Figure 6. Relative NFAT5 target gene mRNA expression in HepG2 cells under NFAT5 overexpression and high-NaCl conditions.**

(A) Relative SLC5A3 mRNA expression in HepG2 cells following transfection with NFAT5 plasmid and/or culture with 50 mM NaCl. (B) Relative SLC6A12 mRNA expression in HepG2 cells under the same in vitro conditions as in (A). (C) Relative AKR1B1 mRNA expression in HepG2 cells under the same in vitro conditions as in (A). For (A), (B), and (C), data are presented as mean SEM (n=3 biological replicates). In vitro control medium contains 110 mM NaCl, while the high-NaCl treatment groups represent additional NaCl supplementation. ****P* < 0.001, *****P* < 0.0001 vs. non-target siRNA control group; ##*P* < 0.01, ###*P* < 0.001 vs. NFAT5 siRNA + 50 mM NaCl group. ns = not significant. Statistical analysis was performed using one-way ANOVA.

**Supplementary Figure 7. Relative NFAT5 target gene mRNA expression in Caki-1 cells under NFAT5 modulation and high-NaCl conditions.**

(A) Relative SLC5A3mRNA expression in Caki-1 cells following transfection with NFAT5 siRNA and/or culture with 50 mM NaCl. (B) Relative SLC6A12mRNA expression in Caki-1 cells under the same in vitro conditions as in (A). (C) Relative AKR1B1mRNA expression in Caki-1 cells under the same in vitro conditions as in (A). ***P* < 0.01, ****P* < 0.001, *****P* < 0.0001 vs. non-target siRNA control group; *P* < 0.001, ####*P* < 0.0001 vs. NFAT5siRNA + 50 mM NaCl group. ns = not significant. For (A), (B), and (C), data are presented as mean SEM n=6 biological replicates, based on visible data points. In vitro control medium contains 110 mM NaCl, while the high-NaCl treatment groups represent additional NaCl supplementation. Statistical analysis was performed using one-way ANOVA.

**Supplementary Figure 8. Modulation of general stress pathways by NFAT5 knockdown under high-NaCl conditions.**

(A) Western Blot (WB) analysis of pERK1/2, total ERK1/2, and HSP70 protein expression in HepG2 cells following NFAT5 siRNA transfection. The experimental groups include Non-Target siRNA ± 50 mM NaCl and NFAT5 siRNA ± 50 mM NaCl. GAPDH was used as a loading control. (B) Quantification of HSP70 protein expression in HepG2 cells, normalized to GAPDH. (C) Quantification of the pERK1/2Total ERK1/2 ratio in HepG2 cells, normalized to GAPDH. (D) WB analysis of pERK1/2, total ERK1/2, and HSP70 protein expression in Caki-1 cells under the same conditions as in (A). Note the observable increase in GAPDH expression in the high-NaCl groups (highlighted). Due to GAPDH upregulation under high-salt stress, HSP70 was used as the loading control for Caki-1 samples. (E) Quantification of the pERK1/2 / Total ERK1/2 ratio in Caki-1 cells, normalized to HSP70. **P* < 0.05 vs. non-Target siRNA control group; ns = not significant. data are presented as mean SEM (n=3 biological replicates). In vitro control medium contains 110 mM NaCl, while the high-NaCl treatment groups represent additional NaCl supplementation. Statistical analysis was performed using t-test or ANOVA where appropriate.

**Supplementary Figure 9. Modulation of ERK1/2 and HSP70 stress pathways by NFAT5 overexpression in HepG2 cells.**

(A) Western Blot (WB) analysis of pERK1/2, total ERK1/2, and HSP70 protein expression in HepG2 cells following NFAT5 plasmid transfection. The experimental groups include control, +50 mM NaCl, NFAT5 plasmid, and NFAT5 plasmid +50 mM NaCl. GAPDH was used as the loading control. (B) Quantification of HSP70 protein expression in HepG2 cells, normalized to GAPDH. (C) Quantification of the pERK1/2/Total ERK1/2 ratio in HepG2 cells, normalized to GAPD. *P < 0.05 vs. control group; ns = not significant. data are presented as mean SEM (n=3 biological replicates). In vitro control medium contains 110 mM NaCl, while the high-NaCl treatment groups represent additional NaCl supplementation. Statistical analysis was performed using t-test or ANOVA where appropriate.
