# Supplementary figures and images for: High salt-induced osmotic stress differentially modulates hepatocellular and renal carcinoma cell proliferation
Source: Front Oncol. 2026 Jan 9;15:1693591. doi: 10.3389/fonc.2025.1693591 (PMC12827174; doi:10.3389/fonc.2025.1693591)

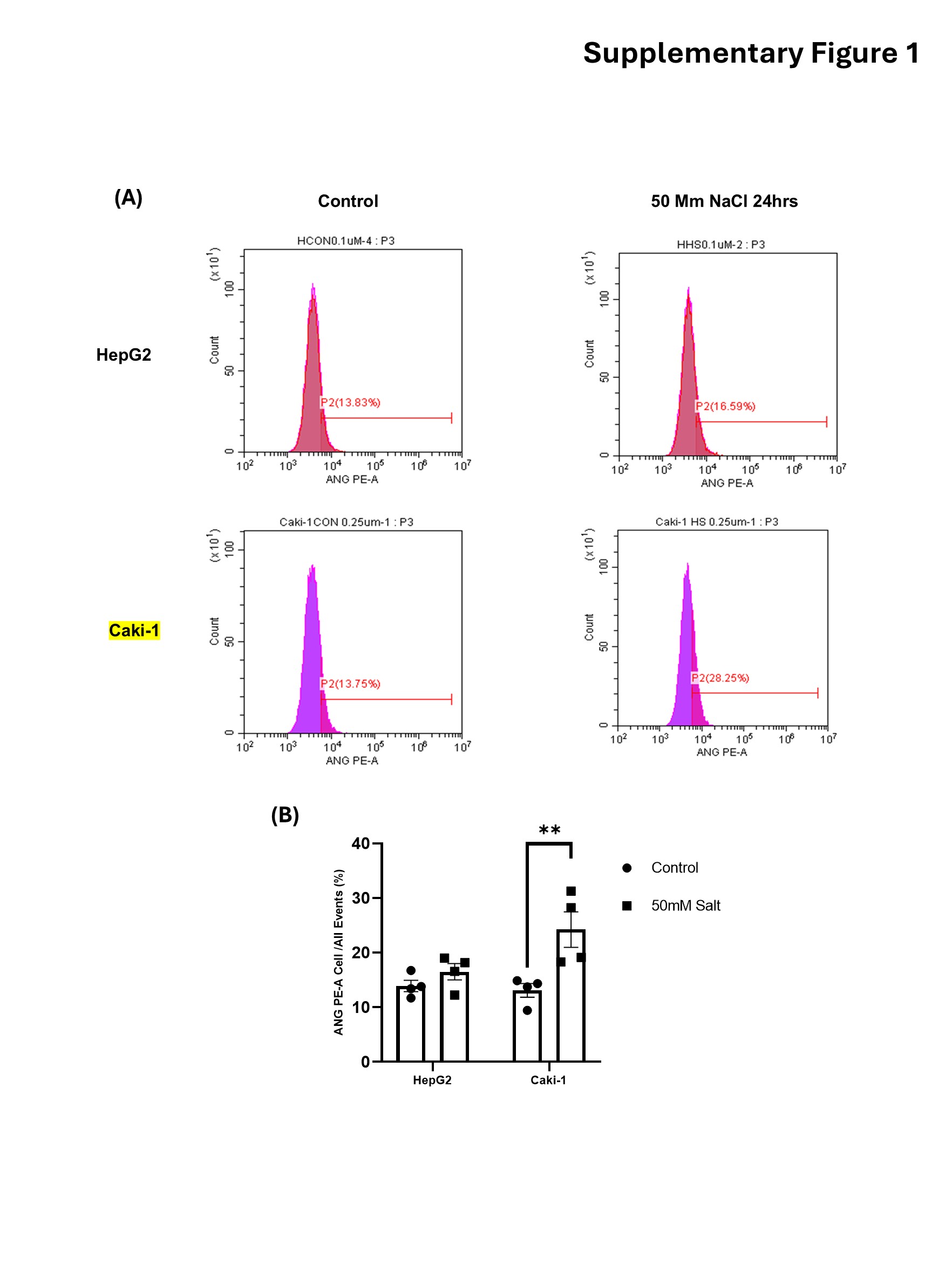

Supplement: Supplementary file 2 [file Image1.jpeg]

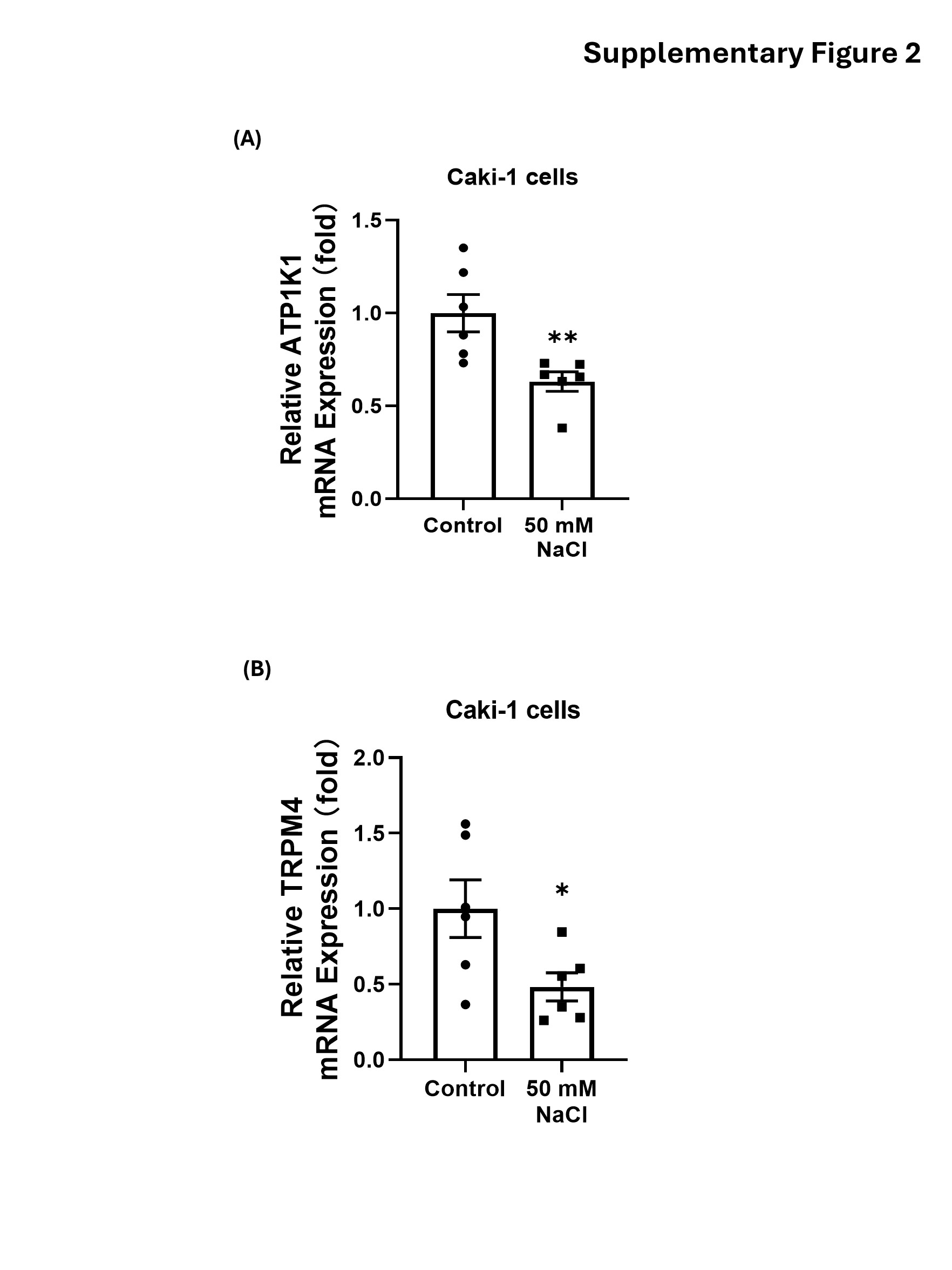

Supplement: Supplementary file 3 [file Image2.jpeg]

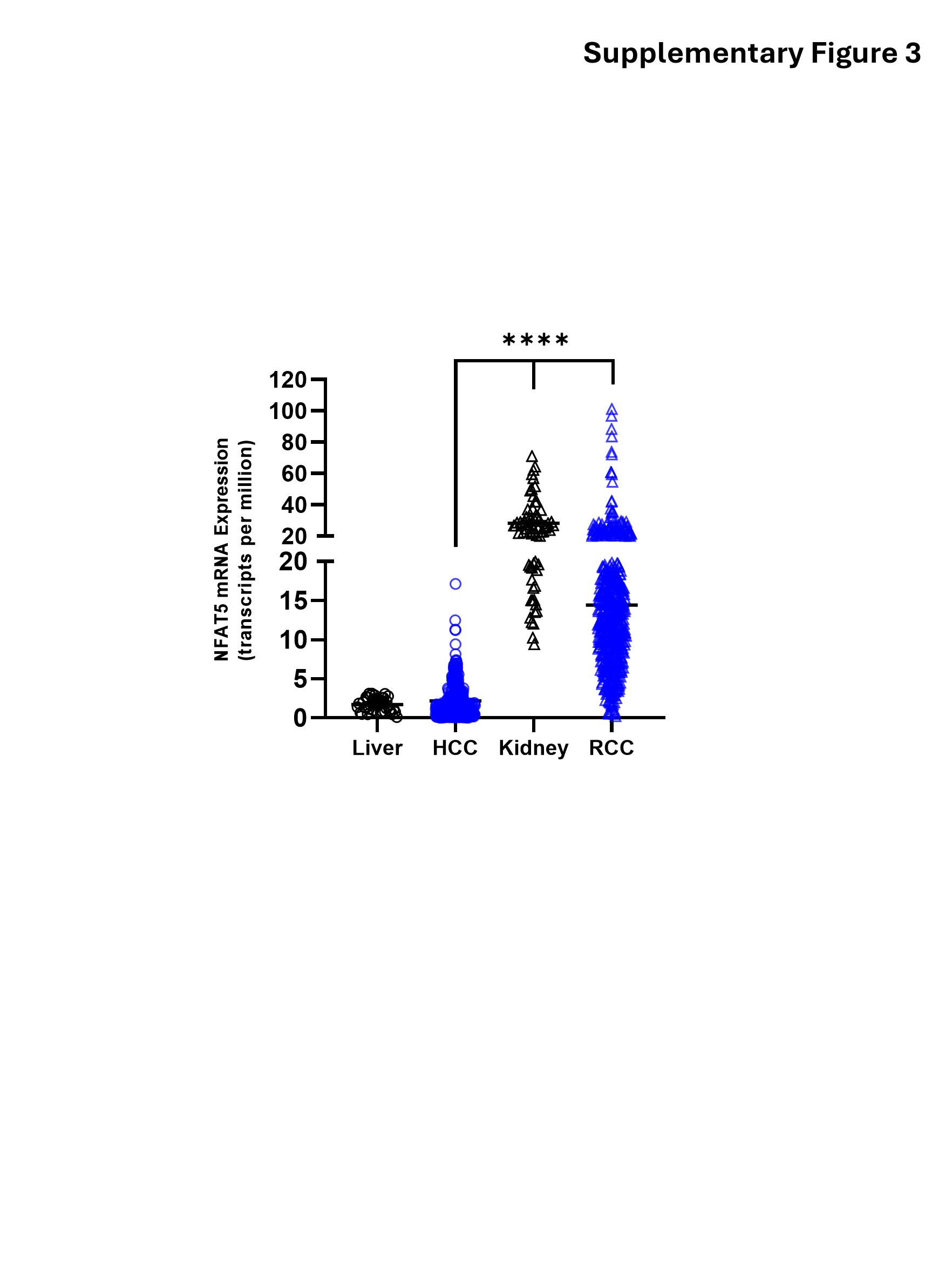

Supplement: Supplementary file 4 [file Image3.jpeg]

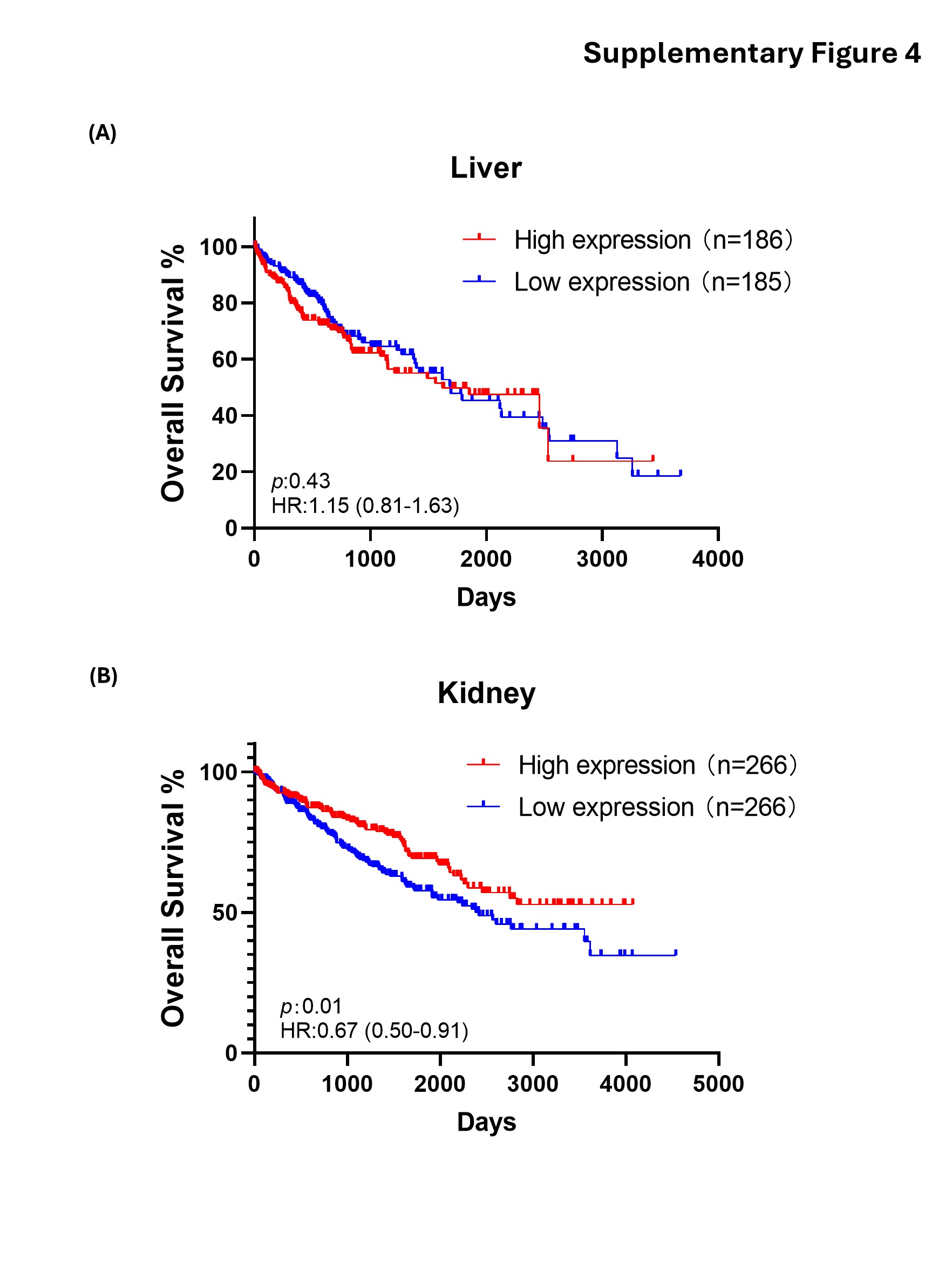

Supplement: Supplementary file 5 [file Image4.jpeg]

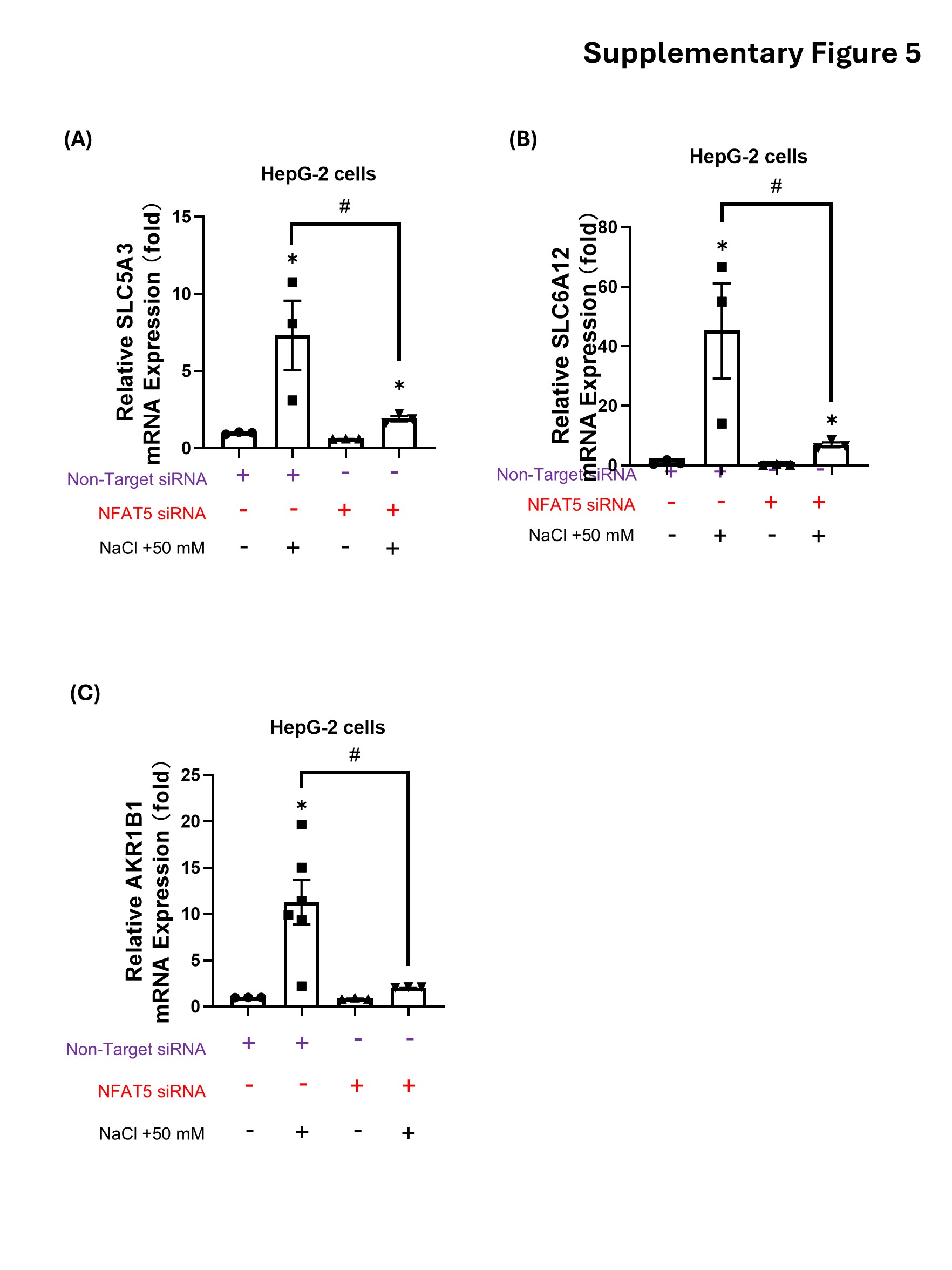

Supplement: Supplementary file 6 [file Image5.jpeg]

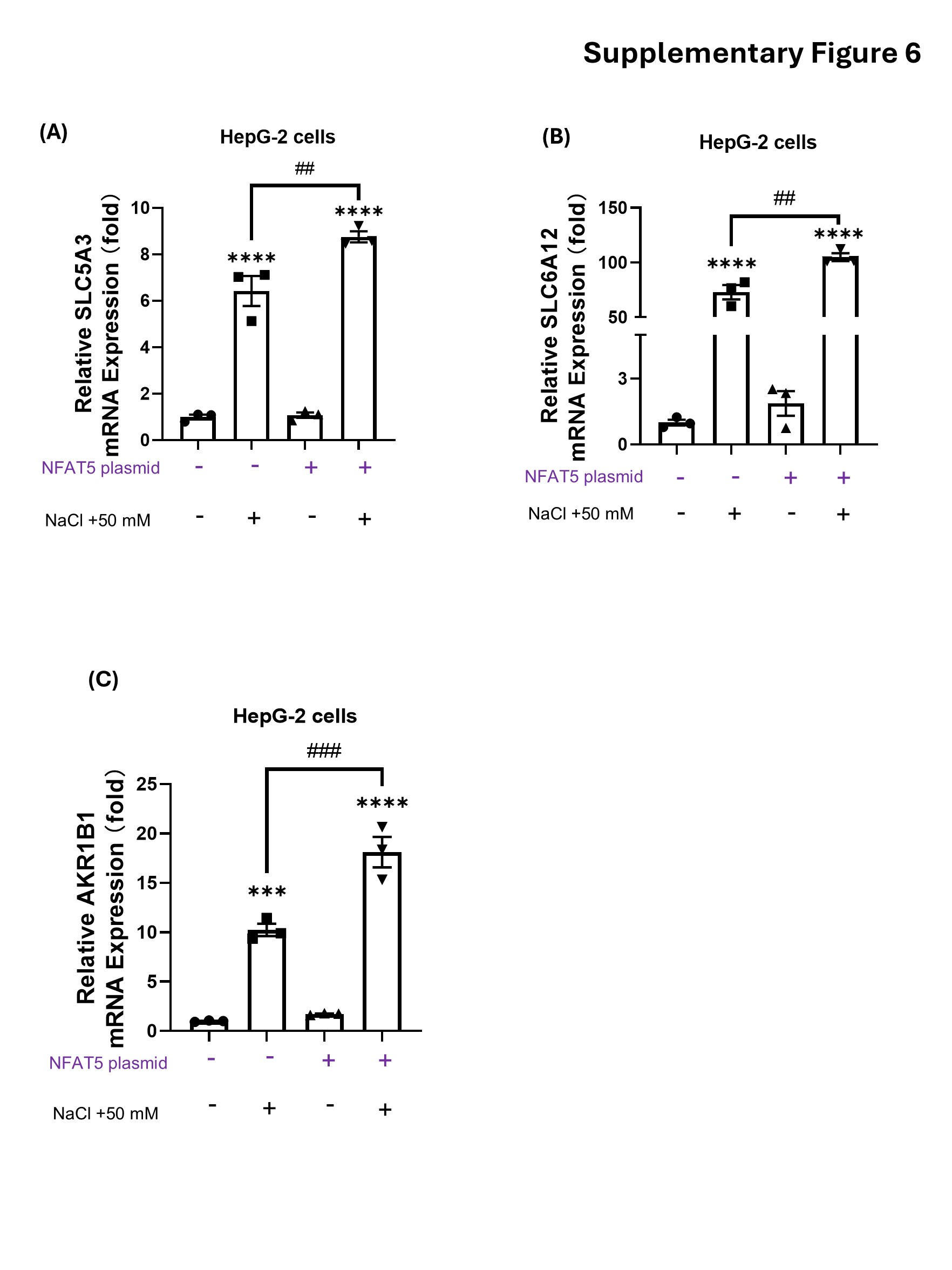

Supplement: Supplementary file 7 [file Image6.jpeg]

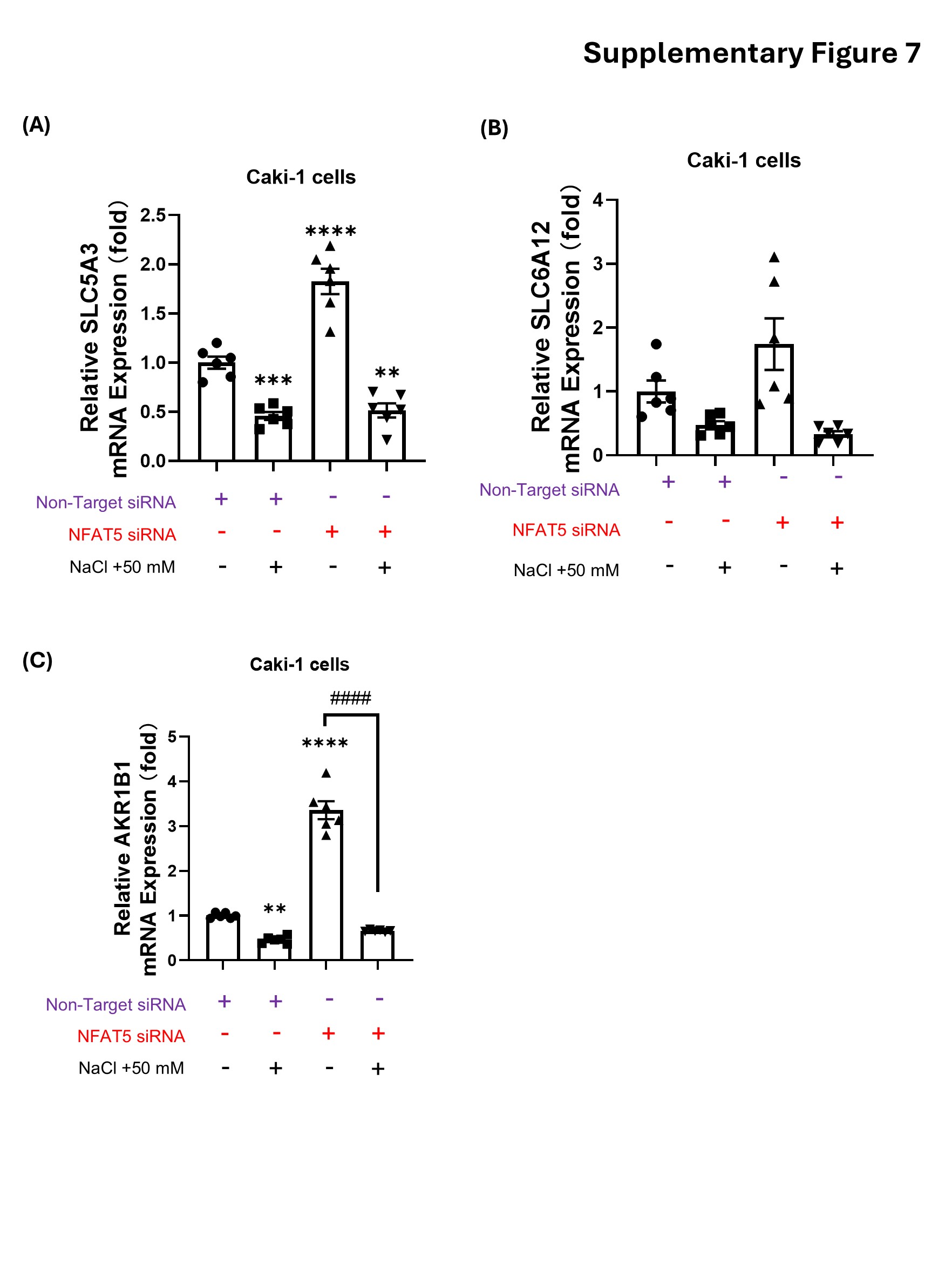

Supplement: Supplementary file 8 [file Image7.jpeg]

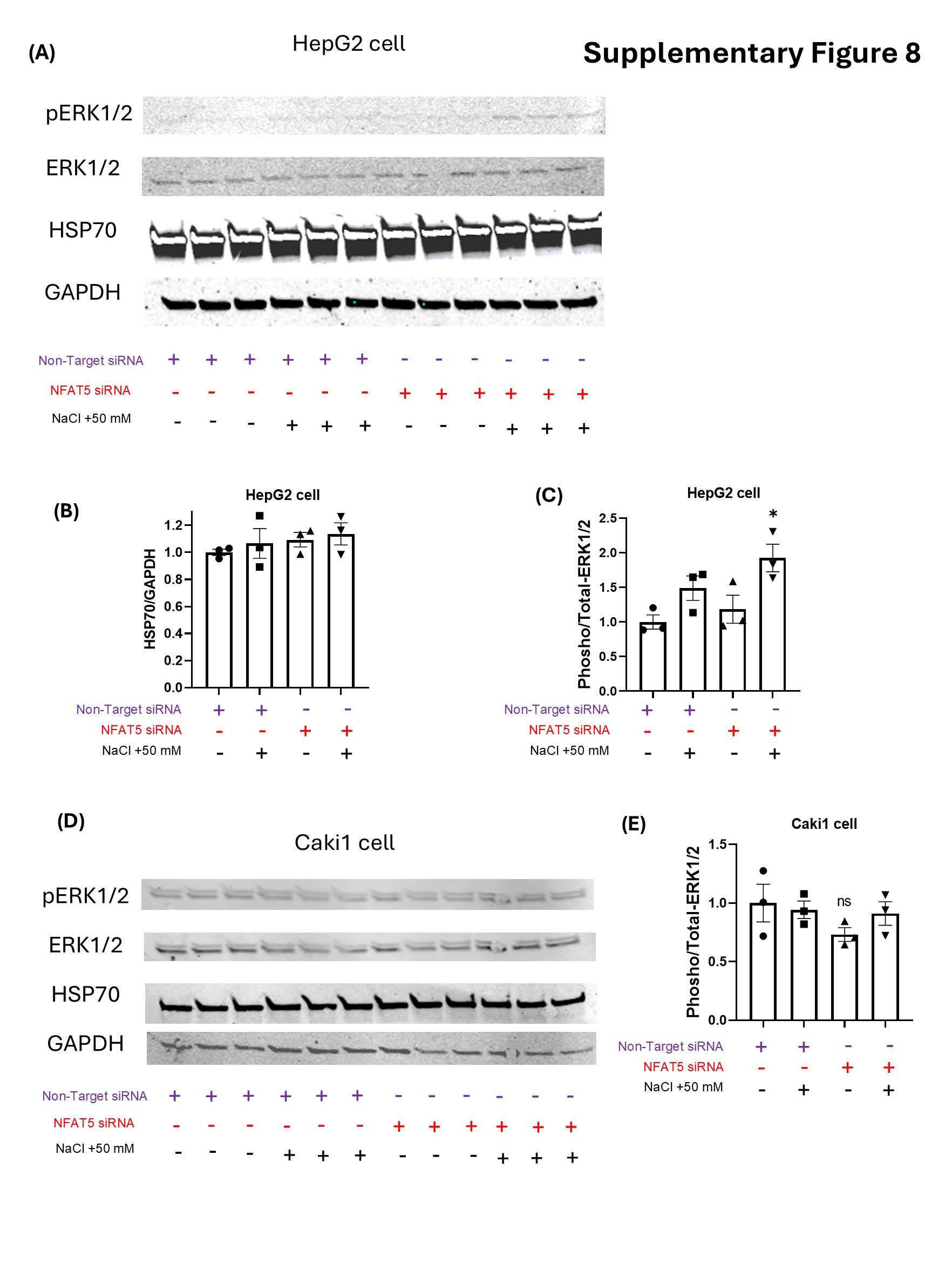

Supplement: Supplementary file 9 [file Image8.jpeg]

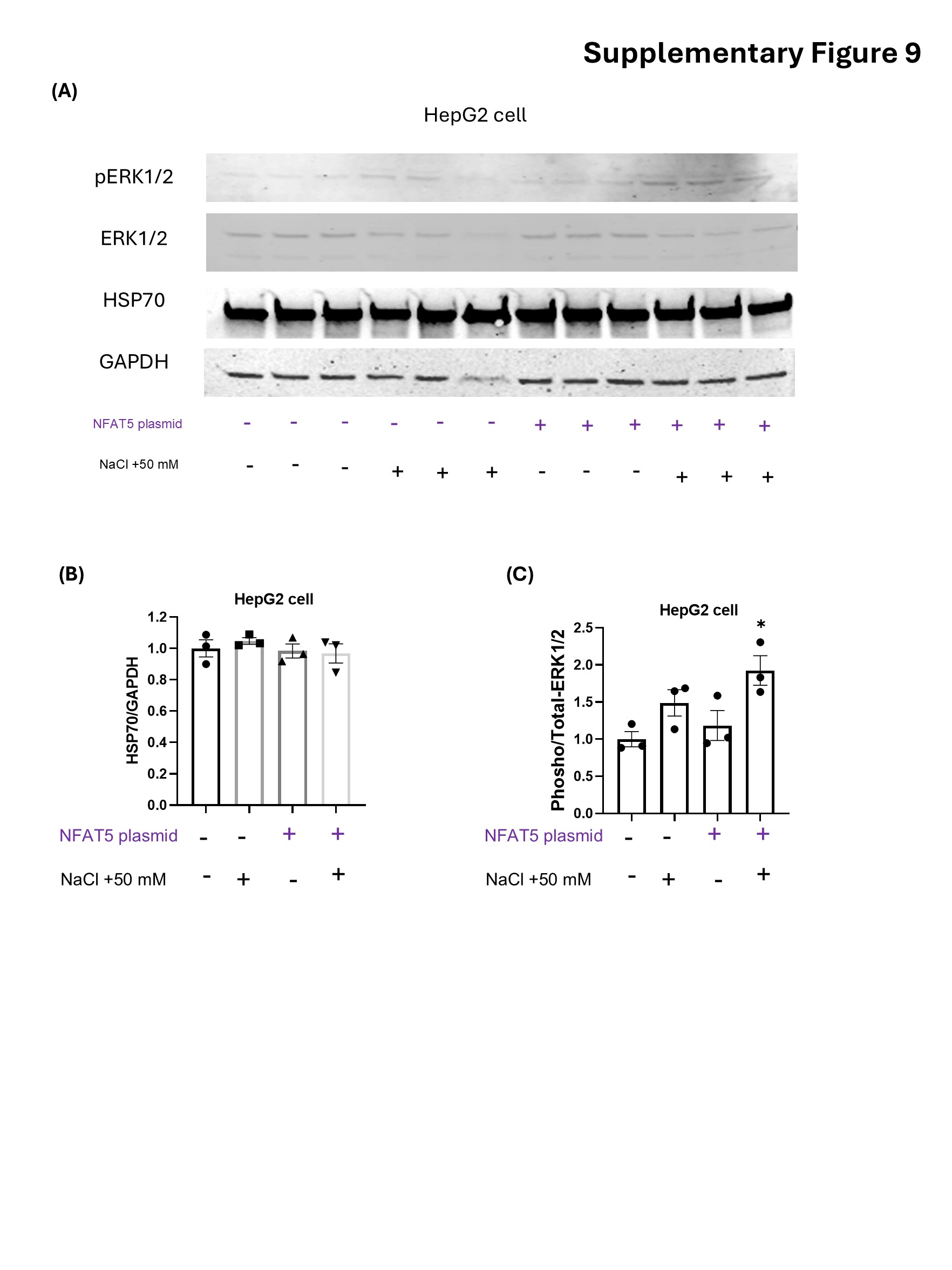

Supplement: Supplementary file 10 [file Image9.jpeg]
